# The impact of gender on early scientific publication and long-term career advancement in Israeli medical school graduates

**DOI:** 10.1186/s12909-021-02598-8

**Published:** 2021-03-17

**Authors:** Limor Y. Tabo, Dan Greenberg, Yosef S. Haviv, Klaris Riesenberg, Lior Nesher

**Affiliations:** 1 Goldman Medical School, Faculty of Health Sciences, Ben-Gurion University of the Negev, Be’er Sheva, Israel; 2 Department of Health Systems Management, School of Public Health, Faculty of Health Sciences, Ben-Gurion University of The Negev, Be’er Sheva, Israel; 3 Infectious Disease Institute, Soroka Medical Center, 1 Rager Street, 84101 Beer-Sheba, Israel

**Keywords:** Gender bias, Interdisciplinary research, Academic success, Professional development

## Abstract

**Background:**

Many medical schools and residency programs incorporate research projects into their curriculum, however most remain unpublished. Little is known on the long-term effect of early-career publication, especially in female graduates.

**Methods:**

We collected data on physicians 15–20 years after graduation (representing a mid-career point), and analysed data on early publication, publication volume and impact according to graduates’ gender and professional characteristics. Physicians were divided into those who never published, early-publishers (EP) who published within 2 years of graduation and late-publishers (LP). We analysed and compared the demographics, publication volume, publication quality as well as current mid-career position.

**Results:**

Of 532 physicians, 185 were EP (34.8%), 220 were LP (41.3%), 127 (23.9%) never published, 491 (92.2%) became specialists and 122 (22.3%) achieved managerial position. Of the 405 who published, the average number of publications was 20.3 ± 33.0, and median (IQR) 9(19). H-index was significantly higher in EP, males, surgical specialists, and those holding a managerial position. Male gender was associated with higher publication rate (OR = 1.742; 95% CI 1.193–2.544; *P* = 0.004). Using quantile regression, female gender was negatively associated with the number of publications in Q50-Q95. Surgical specialty and managerial position were positively associated with publications in Q25 to Q75 and early publication in Q25 and Q75.

**Conclusions:**

We found a strong association between EP and the number, impact, and quality of publications throughout their academic career. This study illuminates the need for further investigations into the causes of gender discrepancies. We should invest in support programs encouraging early high quality research projects for young physicians and female graduates.

**Supplementary Information:**

The online version contains supplementary material available at 10.1186/s12909-021-02598-8.

## Main messages

- We show that early career scientific publication is associated with superior long-term achievements.
- Women publish less than men, both overall as well as early in their career.
- Surgical specialties publish more and earlier than non-surgical (medical) specialties.
- Medical schools and residencies should create tailored programs to support and encourage early publications, especially by women and in non-surgical (medical) specialities.

## Introduction

Early research experience helps foster scientific thought and nurture evidence-based practice in clinical settings [1–5]. Developing research skills is considered to be a significant learning outcome of medical education and is associated with improved short and long-term scientific productivity [6]. In some countries, such as the United States and Germany, physicians can practice medicine without completing a research thesis during their medical school training. Hence, the benefit of completing a medical thesis is more evident for graduates who are pursuing an academic career [7]. However, in many countries, as in Israel, performing an original research project is an integral component of medical students’ education and a formal pre-requisite for obtaining a Medical Doctor (MD) degree as well as part of residency training.

Medical students generally perceive their early research exposure, regardless of whether it is mandatory or an elective process, as a stimulating experience that sparks their research interest and assists in developing scholarly research abilities [8, 9]. However, in recent years there has been a decline in the number of physician-investigators. Despite this decline, there is no uniform strategy within the medical education discipline on how to encourage physicians in training to perform and publish their research. Therefore, more intensive strategies to encourage young physicians to perform clinical research may be needed [1, 2, 10, 11].

Several studies have investigated the scientific impact of training physicians for research and discussed their advantages and limitations. Brancati and colleagues [12] suggested that academic performance and research experience of male students during medical school can predict career achievements in academic medicine 20 years in advance. Riggs et al. [13] showed a positive relationship between early publishing and subsequent publication success among a highly selected group of non-PhD physician-scientists. Agha et al. demonstrated that about one third of medical students in the UK take an intercalated degree, an extra year’s study, to obtain a BSc, BA or BMedSci, with some courses being science oriented [14]. Everee [15] reported that medical professionals in the UK with a BSc degree had a better publication record over ten years compared with academics without a BSc. These studies, however, addressed research-oriented medical students who have actively sought out a research track. A prospective cross-sectional study examined the scholarly products and the career preferences of medical students two years after a mandatory research project course in Sweden [2]. The authors have concluded that the significant outcome of the course is that the scientific collaboration of supervisors and students often continues long after the mandatory research project is completed. However, the proportion of studies performed by medical students resulting in a peer-reviewed journal publication was lower than the average reported in a meta-analysis (an average of 15 and 30%, respectively) [2, 5].

The discrepancies between female and male researchers are well known and are apparent in almost every step of the career of scientists [16]. It has been well described that there is a glass ceiling and gender bias on advancement of women into leadership positions in academic medicine despite no visible barriers [17]. This gap continues despite demographic changes in the trends of the medical workforce, as women outnumber the number of men in many medical schools [18]. As the evidence on the relationship between early publication in women physician’s career and long-term academic performance and achievement is limited, we aimed to investigate this association and to ascertain if medical schools and residency programs should invest more effort in encouraging and supporting women’s research projects during training.

## Methods

### Study population

The study population included all graduates of the Joyce and Irving Goldman Medical School at the Ben-Gurion University of the Negev, who graduated between 1993 and 2003 that were granted a license to practice medicine in Israel. This time frame was chosen as it represents physicians that are currently in their mid-medical practice career point. The Goldman Medical School, established in 1974, is a six-year program that grants a degree of Bachelor of Medical Sciences (B.Med.Sc.) after 3-yr studies and a Doctor of Medicine (M.D.) degree after completion of 6-yr studies and one additional year of rotating internship. All medical schools in Israel require a research thesis as part of the graduation and licensing requirements and nearly all residencies require a 6-month research experience.

### Data collection

We obtained the list of graduates from the School’s records. This list included the graduates name, gender, and year of graduation. We then cross-referenced our list to the public records of active physicians on the Israeli Ministry of Health website which includes their medical practice license and specialty status [19]. This search strategy enabled us to capture possible changes in medical graduates’ surname. For each graduate, we performed a comprehensive search of electronic databases initially using Scopus (Elsevier) and supplementing missing data from Web of Science (WOS), PubMed, and Google Scholar.

We extracted data on the first publication which included title, source (journal), publication date, whether the graduate is the first, last or middle author in the article, and the number of citations. We then used the WOS to collect data on the journal’s impact factor and Journal Citation Report (JCR) rank relevant to the year of the article’s publication or, if this was not available, the impact factor of the earliest recorded year.

We extracted the following information and metrics per graduate: the total number of publications, year of the last publication, overall number of citations, H-index, and number of first/last author publications [20–23]. For each author’s publication, we established whether it was published in a journal ranked as Q1 by the JCR for the year published. An Early publication (EP) was defined as a publication up to 2 years after graduation.

The physicians’ field of specialty was used for analysis according to the following classification: surgical specialty (included all general surgery and all subspecialties including obstetrics and gynaecology) or medical specialty (included general medicine and all subspecialties including family medicine and psychiatry). Finally, we googled the graduate’s name with the aim to obtain information pertaining to the current administrative position: managerial positions were defined as head of any type of medical service or greater (department, division, etc).

### Data analysis

We used χ^2^ tests to determine differences in publication rates according to medical graduates’ characteristics (e.g., gender, year of graduation, specialty). Since the publication data (e.g., number of publications, H-index) were skewed, we calculated and reported median and interquartile range (IQR), in addition to mean and standard deviation values. Comparison of publication and bibliometric data (e.g., journal’s impact factor) were performed using the non-parametric independent-samples Kruskal-Wallis and Mann-Whitney U tests. We used multivariable stepwise binary logistic regression to analyse predictors of EP. Results are reported as odds ratio (OR) values and 95% confidence intervals (CI). We used Spearman’s correlation to assess the bivariate correlations between the H-index and overall number of publications and overall number of citations. Predictors of publication data (number of publications, H-index) were performed using stepwise linear regression (following log-transformation of the data). We report *β* values and 95% CI for each of the significant predictors. We also used quintile regression models and examined the regression coefficients across quintiles Q25, Q50, Q75 and Q95 of the publication data. Quintile regression is a non-parametric method using the median rather the mean value of the data across various quantiles, allowing the comparison of the predictors at different levels of the dependent variables. Data analyses were performed using IBM SPSS Statistics 26 for Windows (Armonk, NY: IBM Corp); *p*-values less than 0.05 were considered statistically significant for all analyses.

## Results

**Fig. 1:**
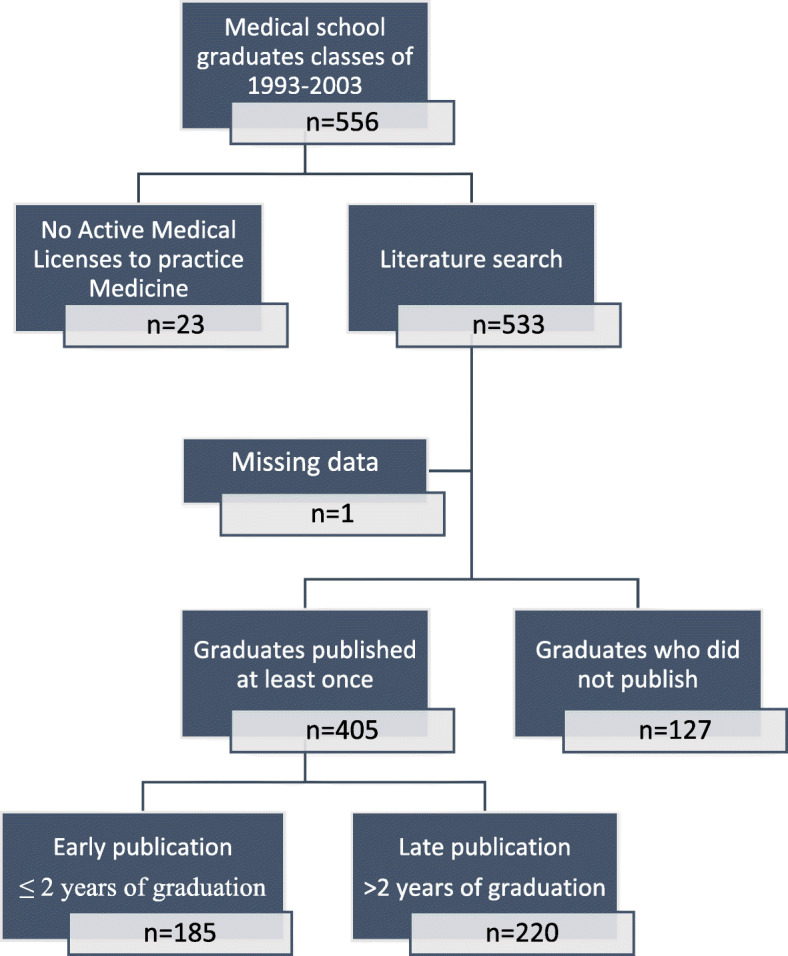
Flow chart of the data extraction on graduates

Of the 556 graduates, analysis was performed on 532; 23 (4.1%) were excluded due to inactive medical license and one due to missing data (Fig. 1).

Approximately 40% of the graduates were female and their proportion among graduates did not differ significantly throughout the study years (Supplementary Table 1A). The baseline characteristics and publication achievements of all 532 graduates included in our analysis are presented in Table 1.

**Table 1:** Baseline characteristics and publication achievements of medical graduates’ classes of year 1993 and 2003 comparing those who never published to those published at least once, and within those who published comparing early vs. late publication

Characteristic	All graduates ***n*** = 532			Published at least once ***n*** = 405		
	Never published (***N*** = 127)	Published at least once (***N*** = 405)	***P***-value	Early publication (***N*** = 185)	Late publication (***N*** = 220)	***P*** value
**Gender**						
Female	60 (28.3%)	152 (71.7%)	0.051	59 (38.8%)	93 (61.2%)	0.032
Male	67 (20.9%)	253 (79.1%)		126 (49.8%)	127 (50.2%)	
**Any specialty**						
Yes	106 (21.6%)	385 (78.4%)	< 0.001	174 (45.2%)	211 (54.8%)	0.391
No	21 (51.2%)	20 (48.8%)		11 (55.0%)	9 (45.0%)	
**Number of specialities**						
0	21 (51.2%)	20 (48.8%)	< 0.001	11(55.0%)	9 (45.0%)	0.761
1	86 (26.3%)	241 (73.7%)		108 (44.8%)	133 (55.2%)	
2	19 (12.1%)	138 (87.9%)		64 (46.4%)	74 (53.6%)	
3	1 (14.3%)	6 (85.9%)		2 (33.3%)	4 (66.7%)	
**Type of speciality**^**a**^						
Surgical	23 (13.1%)	153 (86.9%)	< 0.001	70 (45.8%)	83 (54.2%)	0.982
Medical	104 (29.2%)	252 (70.8%)		115 (45.6%)	137 (54.4%)	
**Managerial position**						
Yes	9 (7.4%)	113 (92.6%)	< 0.001	51 (45.1%)	62 (54.9%)	0.891
No	118 (28.8%)	292 (71.2%)		134 (45.9%)	158 (54.1%)	

^**a**^ A Surgical specialty includes the following: general surgery, OBGYN, urology, orthopaedics, plastic surgery, cardiothoracic surgery, vascular surgery, paediatric surgery, otolaryngology, and ophthalmology

Ninety-two percent of graduates completed at least one specialty training following graduation, 29% completed two specialties and about 1% completed three specialties. Among those completing at least one specialty, 98 (20%) specialized in pediatrics, 79 (16.1%) in obstetrics and gynecology, 77 (15.7%) in internal medicine and 53 (10.8%) in family medicine. The proportion of graduates completing at least one specialty was similar in female and male physicians (92.0 and 92.5%, respectively; *p* = 0.826). The number of specialties among 491 graduates with at least one specialty did not differ between the two genders: 69.7% of female physicians had one specialty and 30.2% two or more specialties while 64.5% of male physicians had one specialty and 35.5% had two or more (*p* = 0.448). Among graduates with at least one specialty, 176 (35.8%) trained in a surgical-type specialty, 315 (64.2%) in a medical/non-surgical type specialty. The proportion physicians with a surgical-type specialty was somewhat higher among males compared with females (35.9% vs. 28.8%, respectively), but these differences were not statistically significant (*p* = 0.086). The type or number of specialties did not differ significantly by graduation year (Supplementary Table 1). The proportion of physicians holding a managerial position was substantially higher among male (28.4%) compared with female physicians (14.6%); *p* < 0.001. Overall, 405 (76.1%) of all graduates published at least once during the study period. Publication rates were higher among physicians with any kind of specialty, among those with a surgical compared with a medical specialty and generally increased with the number of specialties (*p* < 0.001 for all comparisons). Publication rates were also higher among those holding a managerial position (p < 0.001) and tended to be higher among male physicians, although this difference was not statistically significant. The vast majority (90.3%) of female physicians holding a managerial position published at least one paper, compared with those with no managerial position (68.5%); *p* = 0.013. Similar findings were found among male physicians with a publication rate of 93.4% among those holding a managerial position compared with those who did not (73.4%); *p* < 0.001.

### Early publication

Overall, 185 physicians (34.8% of all 532 graduates and 45.7% among those who published at least once) have published an article in the scientific literature within 2-years of graduation. The percentage of EP increased gradually over the years from 21.6% 1993 to 37.3% in 2003 *p* = 0.007 (Supplement Figure 1). Among those who published at least once, the proportion of EP was higher among males compared with females (49.8 and 38.8%, respectively; *p* = 0.032), but all other characteristics were not associated with EP (Table 1). In a multivariable logistic regression analysis, male gender was associated with higher publication rate (OR = 1.742; 95% CI 1.193–2.544; *P* = 0.004), and the EP rate increased in recent years (OR = 1.088; 95% CI 1.026–1.154; *p* = 0.005). The first publication appeared in a wide range of medical and other journals, and various medical fields. Of 185 publications, 162 (87.6%) were published in a journal with an impact factor at the year of publication. Overall, of these publications, 26.5, 23.8, 20.0, and 17.8% were published in Q1, Q2, Q3, and Q4 journals, respectively, with no significant differences between female and male physicians. The mean (±SD) impact factor of the publishing journals was 1.77 (±1.73), and the median (IQR) was 1.04 (1.66) with no significant differences between female and male physicians. Medical graduates were the first authors in 94 (50.8%), second or middle authors in 86 (46.5%) and last authors in 5 (2.7%) of the publications. The proportion of first and last authorship was substantially higher among male compared with female physicians (58.7% vs. 42.4%, respectively; *p* = 0.038). As of 2018, the mean (±SD) number of citations to these publications was 23.99 (±38.24), and the median (IQR) was 11.0 (24.0).

### Publication volume

Of those that published at least one paper (*n* = 405), the average number of articles was 20.3 (±33.0), with a median (IQR) of 9 (19). Table 2 presents the average and the median number of publications by gender, specialty, managerial position, and author’s placement in the first publication.

**Table 2:** Overall publication volume and H-index of 405 medical graduates who published at least once

Variables		Mean of publications per graduate ± SD (95% CI for mean)	Median (IQR) of publications per graduate	***P*** value**
**Gender**	Female	10.70 ± 13.36	6 (11)	< 0.001
	(*n* = 152)	(8.56–12.85)		
	Male	26.11 ± 39.34	12 (29)	
	(*n* = 253)	(21.24–30.98)		
**Specialty**	No specialty	10.50 ± 25.06	4.0 (5)	0.003
	(*n* = 20)	(−1.23–22.23)		
	Any specialty	20.84 ± 33.29	10.0 (19)	
	(*n* = 385)	(17.50–24.17)		
**No. of Specialties**	0	10.50 ± 25.06	4.0 (5)	0.001
	(*n* = 20)	(−1.23–22.23)		
	1	20.05 ± 31.07	9.0 (20)	
	(*n* = 241)	(15.64–24.45)		
	2	21.55 ± 9.79	10.5 (17)	
	(*n* = 138)	(16.32–26.78)		
	3	36.17 ± 25.81	35.0 (44)	
	(*n* = 6)	(11.0–61.33)		
**Type of specialty**^**a**^	Surgical	23.08 ± 36.23	12 (20)	< 0.001
	specialty	(17.30–28.87)		
	(*n* = 153)			
	Medical	18.65 ± 30.80	6.5 (18)	
	specialty	(14.83–22.47)		
	(*n* = 252)			
**Managerial position**	Yes	31.54 ± 43.95	19 (32)	< 0.001
	(*n* = 113)	(23.35–39.73)		
	No	15.99 ± 26.44	7 (12)	
	(*n* = 292)	(12.94–19.03)		
**Author’s placement (first/last) in first publication**	Yes	24.90 ± 39.20	12 (25)	0.01
	(*n* = 192)	(19.32–30.48)		
	No	16.21 ± 25.54	8 (15)	
	(*n* = 213)	(12.76–19.66)		
**H-index**				
**Time of first publication**	Early publication	7.74 ± 7.678	6 (7)	< 0.001
	(*n* = 185)	(6.63–8.85)		
	Late publication	5.37 ± 5.223	4 (5)	
	(*n* = 220)	(4.68–6.07)		
**Gender**	Male	7.67 ± 7.531	6 (8)	< 0.001
	(*n* = 253)	(6.74–8.60)		
	Female	4.43 ± 3.723	3 (4)	
	(*n* = 152)	(3.83–5.02)		
**Graduation year**	1993–2003	9.28 ± 9.584	7 (10)	0.037
	(*n* = 405)	(5.63–12.92)		
	1994	7.28 ± 7.09	5.50 (8)	
	(*n* = 32)	(4.72–9.84)		
	1995	9.47 ± 10.589	5 (11)	
	(*n* = 32)	(5.65–13.29)		
	1996	7.97 ± 6.703	6.50 (10)	
	(*n* = 32)	(5.55–10.39)		
	1997	5.95 ± 4.478	5 (8)	
	(*n* = 37)	(4.45–7.44)		
	1998	6.92 ± 7.54	5 (6)	
	(*n* = 37)	(4.40–9.43)		
	1999	6.09 ± 6.089	4 (4)	
	(*n* = 35)	(3.99–8.18)		
	2000	7.06 ± 6.455	5 (5)	
	(*n* = 31)	(4.70–9.43)		
	2001	4.93 ± 3.489	4 (5)	
	(*n* = 40)	(3.81–6.04)		
	2002	4.62 ± 4.035	3 (5)	
	(*n* = 53)	(3.51–5.73)		
	2003	4.34 ± 3.766	3 (4)	
	(*n* = 47)	(3.23–5.45)		
**Specialty**	Yes	6.52 ± 6.353	5 (7)	0.017
	(*n* = 385)	(5.88–7.15)		
	No	5.25 ± 9.888	2 (4)	
	(*n* = 20)	(0.62–9.88)		
**No. of Specialties**	0	5.25 ± 9.888	2 (4)	0.004
	(*n* = 20)	(0.62–10.82)		
	1	6.21 ± 6.324	4 (6)	
	(*n* = 241)	(5.41–7.01)		
	2	6.83 ± 6.365	5 (7)	
	(*n* = 138)	(5.57–7.90)		
	3	11.67 ± 5.538	12.50 (10)	
	(*n* = 6)	(5.86–17.48)		
**Type of specialty**	Surgical specialty	6.86 ± 6.188	5 (5)	0.007
	(*n* = 153)	(5.87–7.85)		
	Medical specialty	6.29 ± 6.462	4 (7)	
	(*n* = 232)	(5.45–7.12)		
**Managerial position**	Yes	8.57 ± 7.579	6 (9)	< 0.001
	(*n* = 113)	(7.15–9.98)		
	No	5.64 ± 5.935	4 (5)	
	(*n* = 292)	(4.95–6.32)		
**Author’s placement in first publication**	First author	7.15 ± 6.946	5 (8)	NS
	(*n* = 179)	(6,12–8.17)		
	Last author	6.69 ± 7.729	5 (8)	
	(*n* = 13)	(2.02–11.36)		
	Middle author	5.86 ± 6.111	4 (6)	
	(*n* = 213)	(5.03–6.68)		

^a^ A Surgical specialty includes the following: general surgery, OBGYN, urology, orthopaedics, plastic surgery, cardiothoracic surgery, vascular surgery, paediatric surgery, otolaryngology, and ophthalmology

**Mann-Whitney or Kruskal-Wallis tests

In a univariate analysis, the mean and the median total number of publications was significantly lower among female physicians (*p* < 0.001), was higher in graduates with a specialty compared with those with no specialty (p < 0.001), higher among physicians with managerial positions (p < 0.001) and among physicians placed first or last in their first publication (*p* = 0.01). The mean and median number of publications did not differ substantially by year of graduation, despite the longer follow-up time for graduates in earlier years.

Table 3 describes the multivariable stepwise linear regression analysis demonstrating that female gender and graduation year had negative impact on overall publication, while EP, number of specialties, a surgical specialty and managerial position were positive predictors of the number of publications (Adjusted model R^2^ = 0.216). In the quantile regression analysis (Table 4), female gender was negatively associated with the number of publications in Q50 to Q95. Surgical specialty and managerial position were positively associated with publications in Q25 to Q75 and early publication in Q25 and Q75.

**Table 3:** Linear regression: predictors of the number of publications and H-index^**a**^ of 405 graduates who published at least once

	Variables	β	95% CI for mean	*P* value
Overall publication volume	Graduation year	−0.053	− 0.089, − 0.017	0.004
	Female gender	−0.427	− 0.663, − 0.191	< 0.001
	No. of specialties	0.516	0.313, 0.719	< 0.001
	Surgical specialty	0.638	0.392, 0.884	< 0.001
	Early publication	0.434	0.207, 0.662	< 0.001
	Managerial position	0.611	0.355, 0.866	< 0.001
	Adjusted R^2^ = 0.216			
H-index	Graduation year	−0.055	−0.080, − 0.029	< 0.001
	Female gender	−0.303	−0.472, − 0.133	0.001
	No. of specialties	0.308	0.140, 0.433	< 0.001
	Surgical specialty	0.350	0.162, 0.453	< 0.001
	Early publication	0.407	0.239, 0.565	< 0.001
	Managerial position	0.330	0.146, 0.510	< 0.001
	Adjusted R^2^ = 0.197			

^**a**^Overall publication volume and H-index data were transformed using the natural log

**Table 4:** Quantile regression analyses for number of publications and H-index of 405 graduates who published at least once

**Variables**	**Total number of publications** **Quantile Regression Coefficients (*p*-values)**			
	**25th**	**50th**	**75th**	**95th**
Graduation year	0.095 (*p* = 0.233)	−0.294 (*p* = 0.144)	− 0.933 (*p* = 0.070)	**− 5.000 (*p*** **= 0.028)**
Female gender	−0.571 (*p* = 0.360)	**−2.882 (*p*** **= 0.031)**	**−8.333 (*p*** **= 0.015)**	**−37.500 (*p*** **= 0.013)**
No. of Specialties	**1.333 (*p*** **= 0.014)**	**3.235 (*p*** **= 0.005)**	5.667 (*p* = 0.054)	−5.500 (*p* = 0.671)
Surgical specialty	**3.667 (*p*** **< 0.001)**	**5.529 (*p*** **< 0.001)**	**7.133 (*p*** **= 0.044)**	1.000 (*p* = 0.949)
Early publication	**1.190 (*p*** **= 0.049)**	2.294 (*p* = 0.074)	**8.133 (0.014)**	16.000 (0.270)
Managerial position	**2.190 (*p*** **= 0.001)**	**9.000 (*p*** **< 0.001)**	**12.933 (*p*** **< 0.001)**	27.000 (*p* = 0.270)
First or last author	0.429 (*p* = 0.712)	1.000 (*p* = 0.436)	5.600 (*p* = 0.088)	**31.500 (0.030)**
R^2^	0.041	0.075	0.121	0.204
**Variables**	**H-index Quantile Regression Coefficients (*p*-values)**			
	**25th**	**50th**	**75th**	**95th**
Graduation year	**−0.111 (*p*** **= 0.010)**	**−0.250 (*p*** **= 0.001)**	**−0.600 (*p*** **< 0.001)**	**−1.385 (*p*** **< 0.001)**
Female gender	**−0.778 (*p*** **= 0.006)**	**−1.500 (*p*** **= 0.002)**	**−3.000 (p = 0.001)**	**−5.3855 (*p*** **= 0.019)**
No. of Specialties	**0.889 (*p*** **< 0.001)**	**1.500 (*p*** **< 0.001)**	0.900 (p = 0.233)	0.231 (*p* = 0.907)
Surgical specialty	**1.778 (*p*** **< 0.001)**	**1.500 (*p*** **= 0.002)**	0.400 (*p* = 0.660)	0.692 (*p* = 0.770)
Early publication	**1.111 (*p*** **< 0.001)**	**2.000 (*p*** **< 0.001)**	**1.700 (p = 0.044)**	**5.154 (*p*** **= 0.020)**
Managerial position	**1.222 (*p*** **< 0.001)**	**2.250 (*p*** **< 0.001)**	**2.000 (*p*** **= 0.035)**	4.308 (*p* = 0.081)
First or last author	0.0082 (*p* = 1.00)	0.250 (*p* = 0.584)	1.300 (*p* = 0.124)	3.000 (*p* = 0.173)
R^2^	0.067	0.112	0.127	0.225

### Publication quality and impact

The impact of publications was examined using the H-index metric. These data are presented in Table 2. The H-index was higher in those with an EP (*p* < 0.001), in males compared with females (*p* < 0.001), in those who have a specialty compared with those who have not (*p* = 0.017), and in those holding a managerial position (*p* < 0.001). H-index was lower for physicians with a medical specialty (*p* = 0.007) and increased with the number of specialties/sub-specialties culminating as highest for physicians with three specialties (*p* = 0.004). Differences in H-index were also found by year of medical school graduation. This metric did not vary by author’s placement in the first published article (*p* = 0.130).

As with the number of publications, in a multivariable stepwise linear regression analysis, the positive predictors of the H-index metric were surgical specialty, number of specialties, EP, graduation year, managerial position. H-index was negatively associated with female gender and the graduation year (Adjusted model R^2^ = 0.203). In the quintile regression analysis, female gender and graduation year were negatively associated and EP was positively associated with the H-index in all quintiles.

The association of EP and scientific impact at the midpoint of career comparing EP to LP is shown in Table 5, demonstrating that EP was associated with the overall number of publications, citations as well as the H-index.

**Table 5:** The association between early publication and scientific impact at the midpoint of career among 405 graduates who published at least once

Variables		Mean ± Std. deviation (95% CI for mean )	Median (IQR)	***P*** value
**Publications overall amount**	**Early publication**	24.92 ± 39.09	11 (25)	0.01
	(*n* = 185)	(19.25–30.59)		
	**Late publication**	16.46 ± 26.27	8 (15)	
	(*n* = 220)	(12.97–19.95)		
**Citations overall amount**	**Early publication**	511.17 ± 1220.5	164 (338)	< 0.001
	(*n* = 185)	(334.13–688.21)		
	**Late publication**	244.75 ± 474.45	77.5 (190)	
	(*n* = 220)	(181.71–307.79)		
**H-index**	**Early publication**	7.74 ± 7.68	6 (7)	< 0.001
	(*n* = 185)	(6.63–8.85)		
	**Late publication**	5.37 ± 5.22	4 (5)	
	(*n* = 220)	(4.68–6.07)		

## Discussion

Medical training at Ben-Gurion University of the Negev includes a mandatory research project performed in the final years of school. Many medical schools include such a project in their training, and students are encouraged to publish the results of this project. Publication within two years of graduation is considered a successful completion of the research project. In our large cohort, we demonstrated that publishing within two years of graduation was associated with increased professional achievement of physicians at their mid-level career point. This association was demonstrated in all parameters examined including overall publication and citation volumes and H-index as well as obtaining a managerial position. In our cohort, about a third of the students published early, a proportion similar to what has been reported [5]. This practice of encouraging students to publish requires allocation of resources, mostly in faculty time and effort. The faculty members supervising research projects can include clinical mentors as well as full time researchers as shown by Al-Busaidi and colleagues [24]. They demonstrated that clinical mentors are effective as full-time researchers in supervising medical students in terms of degree grade and research output [24]. Our study confirms and strengthens the importance of performing and publishing a research project during medical school or immediately after graduation and medical schools should encourage this practice.

A successful publication of the initial research project early in the physician’s career is probably a springboard that influences future academic and managerial positions. Although only half of 1st and 2nd year medical students believe that research will be of value to their careers, [4] our study suggests that publishing early may contribute to their research and academic career even in the absence of a physician-scientist career. Early publication may pave the way for future academic advancements, productivity, and selection for managerial positions. Furthermore, it is well accepted that developing research abilities is necessary for robust innovations in patient care [2, 5, 25]. Thus, encouraging medical research and publications appears beneficial for patient care.

We have also shown that in the midpoint career stage more men were found to be in managerial positions (28.4% vs 14.6% *p* < 0.001). Moreover, 90.3% of the women that were in managerial position published in similar rates to men. These results support the association of publication and achievement of managerial positions and suggest that one of the possible causes of hampering women on their way to achieve leadership positions may be lack of publishing. Underrepresentation of women in medicine is a well-known historical fact due to active discrimination of women from medical schools and licensing up to the 1960’s [18]. Indiscriminatory laws have been passed over 60 years ago that have shifted the makeup of physician work force, resulting in women being the majority of medical students as well as physicians in many countries. We can still see unexplained discrepancies in the amount of leadership positions filled by women, a gap that should have been bridged by now [17, 26].

In our cohort, we have observed that male graduates publish significantly more than female graduates. These differences in gender publication patterns continue throughout the years. Furthermore, the only factors that were negatively associated with volume of publications and H-index were graduation year and female gender. Female gender is a negative predictive factor even after controlling for other characteristics and was maintained regardless of year of graduation. For women who wish to enter into academic medicine, many obstacles have been described and impact all levels of academics up to and including medical journal leadership [27, 28]. In our study, we can see that these differences continue to haunt the female graduates throughout their medical career. Initiatives and partnerships with female medical students and young professionals to see how they can be helped in developing an academic career need to be institutionalized. The needs of the female students might be different than their male counterparts and this needs to be studied and potential barriers addressed. Mentorship can be helpful in this regard with the caveat that it does not stretch the limits of the already overworked female faculty members, and this mentorship needs to be recognized and rewarded. We suggest that medical schools to address this issue and implement support programs tailored to the needs of female graduates in order to encourage early publishing and foster an academic career.

In our study, we have demonstrated a higher publication volume and higher H-index in favour of physicians in surgical specialties. For the purpose of this study, we have included obstetrics and gynaecology as a surgical speciality. Svider et al. [29] has shown that H-index varies among the different surgical sub-specialties and is potentially impacted by the number of practitioners as well as research emphasis within a field. However, there is a significant difference when compared to the medical speciality, there may be several reasons for this difference. For an accurate evaluation of the difference in H-index between specialties, a scaling method should be performed, however our cohort was too small to perform such an analysis and further research is required.

Our study has some limitations. Our process of extracting the publications’ data from Scopus included some potential for error (e.g., name spelling, change of name due to marriage, etc.). In order to minimize this type of potential error, we crossed referenced the information available on the graduate’s name with several websites as described in the methods section and re-extracted data with other spelling or surname options. We assumed that EP represents publication of the student’s mandatory research projects, although it is possible that the students were involved in other research projects as well. Our outcomes rely upon the timing of event occurrence from graduation to assess career impact. Some graduates may delay their graduation or complete their research project earlier, however these delays are usually limited to one year, thus should not significantly impact our results.

This study set out to examine whether EP has an association with superior career achievements in a non-selected large cohort of MD graduates. Our results suggest, by objective indices, that EP may be a springboard to a productive and successful physicians’ career. Furthermore, we identified that female graduates are at a disadvantaged point compared to male graduates. These results advocate the need for further research into the cultural or social causes of gender bias and ways to modify them. In our opinion, medical schools and residency programs should invest in early publication by means of research projects performed during training and encourage the students to publish their projects early as a first author in a peer-reviewed medical journal, furthermore, there is a need to create programs tailored to encourage female graduates to publish early.

## Supplementary Information

**Additional file 1: Supplementary Table 1.** Baseline characteristic of 532 medical graduates between the year 1993 and 2003-Gender and specialty.

**Additional file 2: Supplement Figure 1.** Percent of graduates who published early by year of graduation.

## Data Availability

This study is based on analysis performed on a large dataset created by the authors. This dataset is being used for further research and as such is not currently publicly available. The full dataset and analysis will be made available from the corresponding author upon reasonable request.
