# Supplementary material for: The impact of gender on early scientific publication and long-term career advancement in Israeli medical school graduates
Source: BMC Med Educ. 2021 Mar 17;21:163. doi: 10.1186/s12909-021-02598-8 (PMC7967994; doi:10.1186/s12909-021-02598-8)
Supplement: Supplementary file 1 — Additional file 1: Supplementary Table 1. Baseline characteristic of 532 medical graduates between the year 1993 and 2003-Gender and specialty. [file 12909_2021_2598_MOESM1_ESM.docx]

**Supplementary Table 1**: Baseline characteristic of 532 medical graduates between the year 1993 and 2003-Gender and specialty

| **Type of specialty (%)*** | | **Number of specialties** | | | |  |  |  |  |
| --- | --- | --- | --- | --- | --- | --- | --- | --- | --- |
| ***Non-surgical specialty(%)*** | ***Surgical specialty (%)***** | ***3+(%)*** | ***2 (%)*** | ***1(%)*** | ***Any specialty % (n)*** | | | ***Male gender % (n)*** | ***Year*** |
| **64.15** | **35.85** | **1.32** | **29.51** | **61.47** | **92.29 (491)** | | | **60.15 (320)** | **All**  **(n=532)** |
| 45.71 | 54.29 | 5.41 | 13.51 | 75.68 | 94.59 (35) | | | 64.86 (24) | **1993** (n=37**)** |
| 58.97 | 41.03 | 2.50 | 25.00 | 70.00 | 97.50 (39) | | | 70.00 (28) | **1994** (n=40**)** |
| 68.29 | 31.71 | 0.00 | 21.74 | 67.39 | 89.13 (41) | | | 52.17 (24) | **1995** (n=46**)** |
| 51.28 | 48.72 | 0.00 | 27.27 | 61.36 | 88.64 (39) | | | 56.82 (25) | **1996** (n=44**)** |
| 71.79 | 28.21 | 2.27 | 20.45 | 65.91 | 88.64 (39) | | | 65.91 (29) | **1997** (n=44) |
| 66.67 | 33.33 | 1.89 | 32.08 | 56.60 | 90.57 (48) | | | 62.26 (33) | **1998** (n=53) |
| 60.00 | 40.00 | 0.00 | 34.09 | 56.82 | 90.91 (40) | | | 65.91 (29) | **1999** (n=44) |
| 81.63 | 18.37 | 0.00 | 48.08 | 46.15 | 94.23 (49) | | | 55.77(29) | **2000** (n=52**)** |
| 67.35 | 32.65 | 1.92 | 30.77 | 61.54 | 94.23 (49) | | | 63.46 (33) | **2001** (n=52**)** |
| 63.16 | 36.84 | 0.00 | 32.26 | 59.68 | 91.94 (57) | | | 56.45 (35) | **2002** (n=62**)** |
| 63.64 | 36.36 | 1.72 | 31.03 | 62.07 | 94.83 (55) | | | 53.45 (31) | **2003** (n=58) |

*Type of specialty among those with one or more specialty only.

** A Surgical specialty includes the following: general surgery, OBGYN, urology, orthopaedics, plastic surgery, cardiothoracic surgery, vascular surgery, paediatric surgery, otolaryngology, and ophthalmology
