# Supplementary material for: The impact of gender on early scientific publication and long-term career advancement in Israeli medical school graduates
Source: BMC Med Educ. 2021 Mar 17;21:163. doi: 10.1186/s12909-021-02598-8 (PMC7967994; doi:10.1186/s12909-021-02598-8)
Supplement: Supplementary file 2 — Additional file 2: Supplement Figure 1. Percent of graduates who published early by year of graduation. [file 12909_2021_2598_MOESM2_ESM.docx]

**Supplement figure 1.** Percent of graduates who published early by year of graduation

p=0.007
